# The longitudinal impact of reinforcement sensitivity on internet addiction among college students: the mediating role of self-control

**DOI:** 10.3389/fpsyt.2023.1298380

**Published:** 2024-01-08

**Authors:** Jinfeng Xue, Ziyi Li, Wei Zhang, Wendi Li, Li Liu, Zhiyou Zhang

**Affiliations:** ^1^School of Psychology, Central China Normal University, Wuhan, China; ^2^School of Nursing, Hunan University of Chinese Medicine, Changsha, China; ^3^Key Laboratory of Adolescent Cyberpsychology and Behavior, Ministry of Education, Wuhan, China; ^4^Xiamen Hubin High School, Xiamen, China; ^5^School of Mechatronic Engineering, Hunan College of Information, Changsha, China

**Keywords:** reinforcement sensitivity, self-control, internet addiction, longitudinal study, college students

## Abstract

**Introduction:**

As the rapid expanding of internet technology, it is necessary to pay attention to the factors that predict Internet addiction. This study aimed to investigate the longitudinal impact of reinforcement sensitivity on internet addiction among college students and the mediating role of self-control.

**Methods:**

The study involves two follow-up assessments with a 5-month interval. 383 college students’ reinforcement sensitivity, self-control, and internet addiction were measured at two-time points.

**Results:**

①The revised Behavioral Approach System (r-BAS) at Time Point 1 (T1) could predict both T1 and Time Point 2 (T2) internet addiction through the complete mediation of T1 self-control. ②The revised Behavioral Inhibition System (r-BIS) at T1, along with the Fight/Flight/Freeze System (FFFS), can predict T1 and T2 internet addiction through the partial mediation of T1 self-control.

**Conclusion:**

Reinforcement sensitivity can predict current and future internet addiction, with self-control playing a mediating role. This study provides longitudinal experimental evidence for the revised Reinforcement Sensitivity Theory (r-RST), further revealing the underlying mechanisms through which reinforcement sensitivity influences internet addiction. Additionally, it has implications for clinical intervention.

## 1 Introduction

College students experience increased free time and a lack of supervision, leading to a heightened demand for autonomy. Many students struggle to adapt to this change and, as a result, choose to spend their time online, making them highly susceptible to internet addiction (1, 2). Research indicates that the detection rate of internet addiction among college students is as high as 13.6% (3, 4), making it a significant factor affecting the mental and social well-being of college students (5). Therefore, paying attention to the psychological and social adaptation of individuals with internet addiction among the college student population holds crucial practical significance.

## 2 Reinforcement sensitivity and internet addiction

Internet addiction is a behavioral addiction disorder characterized by an inability to control the impulse to use the internet, leading to persistent immersion in online activities [Young (6)]. Internet addiction is associated with a range of emotional and behavioral disorders (7–9). The Interaction of Person-Affect-Cognition-Executive Model (I-PACE Model) posits that personality traits such as reinforcement sensitivity are among the factors influencing an individual’s susceptibility to internet addiction. Specifically, individuals with a reward-oriented and conflict-avoidant trait stemming from their reinforcement sensitivity may be more attracted to internet features such as anonymity, novelty, and immediate feedback. This attraction can lead to the development of maladaptive cognitive perceptions, wherein the online world is perceived as superior to the real world, ultimately culminating in internet addiction (10, 11). In other words, individual reinforcement sensitivity may serve as a predictor for internet addiction.

Reinforcement Sensitivity Theory (RST) is a personality theory based on neurobiology [cited in (12–14)]. This theory categorizes reinforcement sensitivity into the Behavioral Approach System (BAS), Behavioral Inhibition System (BIS), and Fight/Flight System (FFS). The BAS emphasizes individuals’ sensitivity to reward stimuli, manifesting in approach behavior and positive emotional experiences closely associated with impulsivity, adventurousness, and sensation-seeking traits (14, 15). The BIS and FFS, on the other hand, are sensitive to aversive stimuli, both conditional (such as punishment cues or the disappearance of rewards) and unconditional (such as internal pain stimuli) (16). Measurements of reinforcement sensitivity based on the original RST (o-RST) often do not differentiate between BIS and FFS, combining them into a single BIS score (17).

Subsequently, Gray and McNaughton (16) revised the theory. In the revised Reinforcement Sensitivity Theory (r-RST), the revised Behavioral Approach System (r-BAS) remained unchanged, while FFS was transformed into the Fight/Flight/Freeze System (FFFS). FFFS is sensitive to all aversive stimuli, and its activation may lead to confrontational, evasive, or immobilized responses, typically accompanied by emotions such as fear and anxiety (13, 18). On the other hand, the revised Behavioral Inhibition System (r-BIS) detects and resolves conflicts in the environment, often associated with feelings of anxiety (12). From this perspective, activating r-BIS depends on the cooperation of r-BAS and FFFS, forming a conflict between approach and avoidance (16).

A substantial body of research indicated a close association between reinforcement sensitivity and internet addiction (17, 19–21). However, previous research results have yet to be entirely consistent. The main findings can be categorized as follows:

- Some studies indicated that the BAS, rather than the BIS, could predict internet addiction (17, 20).
- Other research suggested that only BIS could predict internet addiction (22).
- Some studies found both BAS and BIS were associated with internet addiction (19, 21).

Despite the variations in research results, based on the above studies, we lean towards the belief that all subsystems of reinforcement sensitivity are connected to internet addiction.

However, the aforementioned studies are all based on cross-sectional research, making it challenging to establish causal relationships. Currently, there is only one longitudinal study examining the predictive effect of BAS and BIS on internet addiction 1 year later. This study found that only BAS could predict the incidence of internet addiction 1 year later, while BIS did not exhibit this predictive effect (22). The researchers suggested that the non-significant predictive effect of BIS might be due to changes in adolescents’ personality traits, exhibiting a tendency to regress toward the mean (or normal). As college students, being adults with relatively stable personalities, it is suggested, based on previous research, that BIS might have delayed predictive effects similar to BAS (21, 22). Additionally, Yen et al. (22) was based on o-RST and did not differentiate between BIS and FFS. Therefore, this study aims to explore the predictive roles of each system of reinforcement sensitivity in internet addiction through a longitudinal approach based on r-RST.

Furthermore, in studies investigating the impact of reinforcement sensitivity on internet addiction, there has been limited consideration of the mediating effects. Existing research has primarily focused on the mediating role of emotions, such as anxiety and depression. For example, Fayazi and Hasani (17) validated the mediating role of anxiety and depression in the relationship between r-BAS, r-BIS, FFFS, and internet addiction. Below we will describe the specific mechanisms that might explain how the r-BAS, r-BIS and FFFS might impact on the internet.

### 2.1 The mediating role of self-control

In addition to depression and anxiety, self-control may also play a mediating role in the relationship between reinforcement sensitivity and internet addiction among college students. Research indicated a correlation between BAS, BIS, and impulsivity or self-control (23, 24). Bacon et al. (25) investigated the predictive role of reinforcement sensitivity on self-control separately based on o-RST and r-RST. Results from the o-RST-based study showed that BAS Drive positively predicted self-control, while BAS Reward Responsibility and BIS negatively predicted self-control. Similarly, results from the r-RST-based study showed that r-BAS Reward-Interest and r-BAS Goal-Drive Persistence positively predicted self-control, while r-BAS Reward-Reactivity, r-BIS, and FFFS negatively predicted self-control. In this study, different dimensions of BAS and r-BAS had varying predictive effects on self-control. However, the study did not explore the predictive direction of the total BAS score on self-control. Previous research indicates that individuals with internet addiction exhibit high BAS and BIS and low self-control characteristics compared to normal groups (9, 19). Based on this research, we hypothesize that in this study, r-BAS, r-BIS, and FFFS will all negatively predict self-control.

Theories related to internet addiction, such as the Dual Process Theory (26, 27) and the I-PACE theoretical model (10, 11), emphasize the crucial role of self-control in the occurrence, development, and maintenance of internet addiction. Abundant research evidence indicated impaired inhibitory control in individuals with internet addiction (9, 28). Recently, Kräplin et al. (29) provided evidence supporting this notion through a longitudinal tracking study, revealing that levels of inhibitory control effectively predict individuals’ future online gaming addiction—lower inhibitory control levels correspond to increased future time spent playing games. Therefore, self-control might act as a mediator in the predictive relationship between reinforcement sensitivity and internet addiction.

In summary, there was currently no study that investigated the impact of reinforcement sensitivity on current and future internet addiction based on r-RST, as well as the mediating role of self-control in this relationship. This study employed a short-term tracking approach to examine the immediate and delayed predictive effects of reinforcement sensitivity on internet addiction and the mediating mechanism of self-control. Based on the aforementioned previous research, we hypothesize:

H1: r-BAS, r-BIS and FFFS have positive predictive effects on current and future internet addiction.

**Figure 1:**
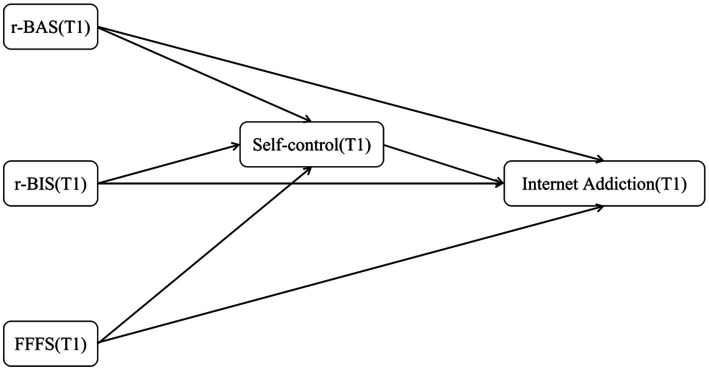
Research model (Immediate).

**Figure 2:**
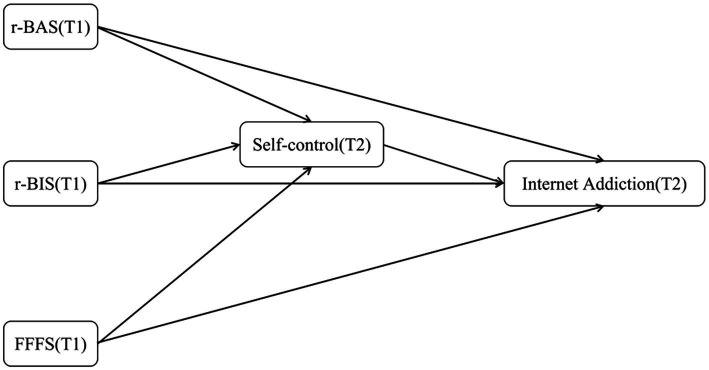
Research model (Delayed).

H2: r-BAS, r-BIS and FFFS negatively predict self-control, and self-control further negatively predicts current and future internet addiction (see Figures 1, 2)

## 3 Method

### 3.1 Participants and procedure

The study employed a cluster sampling approach, distributing questionnaires to two universities in Hubei province. Two follow-up assessments were conducted at intervals of 5 months. In the first assessment in December 2015 (T1), 461 valid questionnaires were collected after excluding invalid responses due to data missing and excessive patterned responding. The second assessment in April 2016(T2) was conducted following the same procedures. Due to factors such as student internships, social activities, and changes in the faculty involved in the supplementary research, 383 participants completed both assessments, resulting in a follow-up rate of 83%. The follow-up rate satisfied the general requirements for structural equation modeling with a sample size ranging from 200 to 400 (30).

We performed data analysis on two occasions, initially in May 2016 and subsequently in August 2023. The results from both instances were consistent. Among the participants were 89 males (23.2%), with an average age of 19.78 years (SD = 1.23). To assess potential biases, t-tests were conducted between participants lost to follow-up at T2 and those included in the final analysis at T1 regarding scores on the Reinforcement Sensitivity Theory (RST) systems and internet addiction. The results indicated no significant differences between the two groups in r-BAS (*t* = 1.025, *p* = 0.308), r-BIS (*t* = 1.370, *p* = 0.174 > 0.05), and FFFS (*t* = −1.048, *p* = 0.297 > 0.05), suggesting that the loss to follow-up did not introduce bias to the study results.

### 3.2 Instruments

#### 3.2.1 The Chinese internet addiction scale

The Chinese Internet Addiction Scale (CIAS) developed by (31) was adopted to measure internet addiction (20). The scale consists of 26 items organized into five dimensions and employs a 4-point Likert scale (1 representing “strongly disagree” and 4 representing “strongly agree”). Higher scores indicate a higher level of internet addiction. In this study, the two assessments’ internal consistency coefficients (Cronbach’s alpha) were 0.92 and 0.94, respectively.

#### 3.2.2 Reuter and Montag’s rRST-Q

Reuter and Montag’s rRST-Q was developed by Reuter et al. (30, 32). The scale consists of 31 items and employs a 4-point Likert scale. After reversing scoring negative items, higher scores on each dimension (r-BAS, r-BIS, and FFFS) indicate stronger sensitivity (32). The internal consistency coefficients for each dimension ranged between 0.75 and 0.78. In this study, we used the naming convention from the questionnaire, i.e., r-BAS, r-BIS, and FFFS. The internal consistency coefficients for the two assessments were 0.73 and 0.79, respectively. The fit indices from the confirmatory factor analysis were as follows: χ^2^/df = 1.98, RMSEA = 0.04, CFI = 0.91.

#### 3.2.3 The self-control scale

The Self-Control Scale (SCS), revised by Tan and Guo (33), was employed in the study. The scale comprises 19 items divided into five dimensions and employs a 5-point Likert scale. Higher scores indicate stronger self-control abilities. The internal consistency coefficients for the scale in the pre-and post-assessment were 0.79 and 0.84, respectively.

## 4 Result

### 4.1 Common method bias test

The Common Method Bias test was conducted using Harman’s Single-Factor Test method. The results indicated 21 and 22 eigenvalues greater than 1 in the two assessments. The variance explained by the first factor was 17.33 and 17.867%, respectively, both of which were less than the critical standard of 40%. These results suggested no significant common method bias was present (18).

### 4.2 Correlation between variables and testing for normality of data

Skewness and kurtosis values were employed to assess the normality of the data. The results indicated that the skewness and kurtosis values for all study variables were below 2, suggesting an approximate normal distribution (Hair, 2010) (Table 1).

**Table 1:** Descriptive results.

Variable	Skewness	Kurtosis	M	SD	1	2	3	4	5	6	7
1. T1 rBAS	**0.02**	**0.54**	**20.34**	**2.20**	**—**						
2. T1 rBIS	**−0.38**	**0.23**	**28.33**	**3.49**	**0.77**	**—**					
3. T1 FFFS	**−0.34**	**0.46**	**27.70**	**3.04**	**−0.09**	**0.39** ^***^	**—**				
4. T1 Self Control	**−0.03**	**−0.10**	**60.45**	**8.76**	**−0.19** ^***^	**−0.42** ^***^	**−0.33** ^***^	**—**			
5. T1 Internet Addiction	**−0.19**	**0.89**	**56.10**	**10.16**	**0.18** ^***^	**0.39** ^***^	**0.42** ^***^	**−0.62** ^***^	**—**		
6. T2 Self Control	**−0.33**	**0.60**	**61.02**	**8.92**	**−0.18** ^***^	**−0.32** ^***^	**−0.27** ^***^	**0.73** ^**^	**−0.46** ^***^	**—**	
7. T2 Internet Addiction	**−0.16**	**1.21**	**54.73**	**10.67**	**0.25** ^**^	**0.35** ^***^	**0.32** ^**^	**−0.50** ^***^	**0.74** ^***^	**−0.60** ^***^	**—**

r-BAS, r-BIS, FFFS represent Behavioral Activation System, Behavioral Inhibition System, and Fight/Flight/Freeze System, respectively; T1, T2 indicate Time Point 1 and Time Point 2, respectively; *N* = 383, ^*^p < 0.05, ^**^p < 0.01, ^***^p < 0.001. The same applies throughout.

Correlation analysis of the main variables between the two assessments revealed that r-BAS, r-BIS, and FFFS at T1 were significantly negatively correlated with self-control at T1 and T2 and positively correlated with internet addiction at T1 and T2. Additionally, self-control at T1 was significantly negatively correlated with internet addiction at T1 and T2, and self-control at T2 was significantly negatively correlated with internet addiction at T2 (Table 1).

### 4.3 Mediation analysis for immediate prediction model of reinforcement sensitivity on internet addiction

Using Model 4 from the PROCESS macro program developed by Hayes, we tested the mediating effects between T1 Reinforcement Sensitivity subsystems and T1 internet addiction. After controlling for gender variables, regression analysis results showed that T1 internet addiction was significantly positively predicted by T1 r-BAS (β = 0.17, *p* < 0.001), T1 r-BIS (β = 0.26, *p* < 0.001), and T1 FFFS (β = 0.32, *p* < 0.001).The model fit index results are as follows: χ2 = 0.004, df = 1, CFI = 1, TLI = 1, SRMR = 0.001. indicating a good fit of the model to the data.

**Figure 3:**
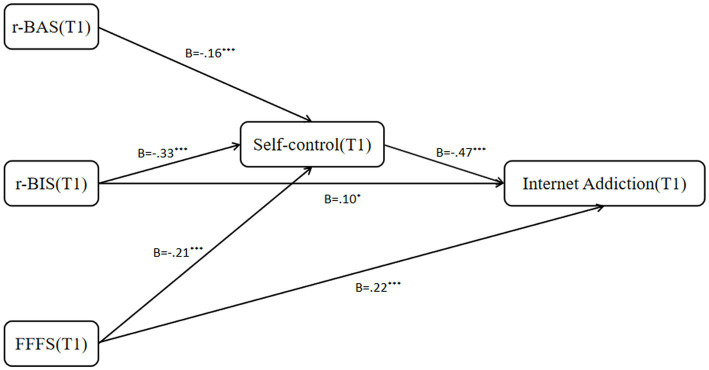
Confirmed research model (Immediate).

The results of the Bootstrap method for testing the mediating effects indicated that the direct effect of T1 r-BAS on T1 internet addiction was not significant. However, the mediating effect was significant [*β* = 0.10, 95%CI (0.04, 0.17)]. T1 r-BIS and T1 FFFS showed significant direct[r-BIS: *β* = 0.17, 95%CI (0.09, 0.26); FFFS: *β* = 0.24, 95%CI (0.16, 0.32)] and indirect effects[r-BIS: *β* = 0.23, 95%CI (0.17, 0.29); FFFS: *β* = 0.17, 95%CI (0.11, 0.23)] on T1 internet addiction. These result indicated that T1 r-BIS and FFFS can directly and indirectly predict T1 internet addiction, while r-BAS can only predict T1 internet addiction through the mediating factor of self-control (Table 2; Figure 3).

**Table 2:** Bootstrap analysis of mediation effects in immediate prediction model (M1).

Effect type	Pathway	Standardized path coefficients	Effect Size	95% Confidence Interval	
				Lower	Upper
Direct Effect	T1rBAS → T1 Internet Addiction	0.06	37.50%	−0.02	0.15
	T1rBIS → T1 Internet Addiction	0.17	42.50%	0.09	0.26
	T1FFFS → T1 Internet Addiction	0.24	57.50%	0.16	0.32
Indirect Effect	T1rBAS → T1 Self Control→T1 Internet Addiction	0.10	60.50%	0.04	0.17
	T1rBIS → T1 Self Control→T1 Internet Addiction	0.23	57.50%	0.17	0.29
	T1 FFFS→T1 Self Control→T1 Internet Addiction	0.17	42.50%	0.11	0.23

Boot CI with upper and lower limits excluding 0 indicates statistical significance.

### 4.4 Mediation analysis for delayed prediction model of reinforcement sensitivity on internet addiction

We used Model 4 from the PROCESS macro to examine the Research Model (Delayed). After controlling the influence of gender, regression analysis results showed that T2 internet addiction was significantly positively predicted by T1 r-BAS (β = 0.13, *p* < 0.01), T1 r-BIS (β = 0.26, *p* < 0.001), and T1 FFFS (β = 0.23, *p* < 0.001). When T2 self-control was included in the equation, the direct predictive effect of T1 r-BAS was insignificant, but the predictive effects of T1 r-BIS and T1 FFFS remained significant (see Table 3). The model fit index results are as follows:χ^2^ = 3.245, df = 1, CFI = 0.99, TLI = 0.932, SRMR = 0.02. These values also suggest a good fit of the model to the data.

**Table 3:** Bootstrap analysis for mediation effects in delayed prediction model (M2).

Effect type	Pathway	Standardized path coefficients	Effect size	95% Confidence interval	
				Lower	Upper
Direct effect	T1rBAS → T1 Internet Addiction	0.04	30.77%	−0.05	0.12
	T1rBIS → T1 Internet Addiction	0.18	51.43%	0.10	0.27
	T1FFFS → T1 Internet Addiction	0.17	54.84%	0.09	0.26
Indirect effect	T1rBAS → T1 Self Control→T1 Internet Addiction	0.09	69.23%	0.04	0.16
	T1rBIS → T1 Self Control→T1 Internet Addiction	0.17	48.57%	0.11	0.24
	T1 FFFS→T1 Self Control→T1 Internet Addiction	0.14	45.16%	0.08	0.20

Boot CI with upper and lower limits excluding 0 indicates statistical significance.

**Figure 4:**
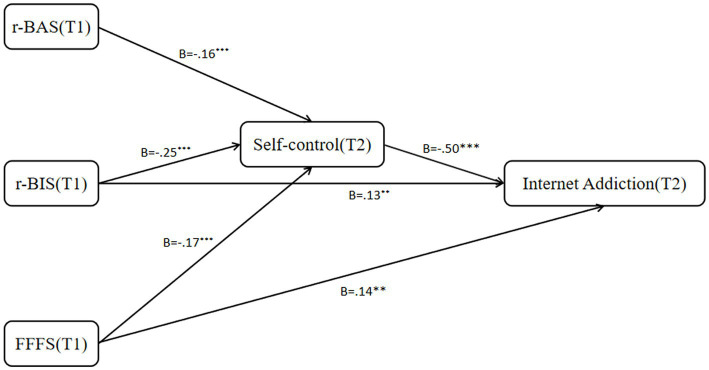
Confirmed research model (Delayed).

The results showed that the direct effect of T1 r-BAS on T2 internet addiction was insignificant. However, the mediating effect was significant [β =0.09, 95%CI (0.04, 0.16)]. T1 r-BIS and T1 FFFS showed significant direct [r-BIS: *β* = 0.18, 95%CI (0.10, 0.27); FFFS: *β* = 0.17, 95%CI (0.09, 0.26)] and indirect effects [r-BIS: *β* = 0.17, 95%CI (0.11, 0.24); FFFS: *β* = 0.14, 95%CI (0.08, 0.20)] on T2 internet addiction. These result indicated that T1 r-BIS and FFFS can directly and indirectly predict T1 internet addiction, while r-BAS can only predict T2 internet addiction through the mediating factor of self-control (Table 3; Figure 4).

## 5 Discussion

This study, based on r-RST and utilizing a tracking approach, explored the predictive effects of reinforcement sensitivity on current and future internet addiction while examining the mediating role of self-control. The research findings indicate that all three subsystems of reinforcement sensitivity could predict current and internet addiction after a 5-month interval. However, the predictive paths of each subsystem to internet addiction were somewhat different. Specifically, r-BAS predicted current and internet addiction after 5 months through the complete mediation of self-control, while r-BIS and FFFS predicted current and internet addiction after 5 months through the partial mediation of self-control.

Consistent with previous research (17, 20, 21), r-BAS, r-BIS, and FFFS predicted individuals’ current internet addiction. Additionally, these subsystems exhibited a long-term impact on internet addiction, as all three could predict internet addiction after 5 months [Yen et al. (22)]. This result indicates that individuals with higher levels of r-BAS, r-BIS, and FFFS are at an increased risk of internet addiction both in the present and the future, confirming a causal relationship between reinforcement sensitivity and internet addiction.

The direct effect of r-BAS was found to be nonsignificant, and it could only indirectly predict internet addiction through the mediation of self-control. The results validated that r-BAS could predict internet addiction through the complete mediation of self-control, aligning with the findings of Park et al. (21) and Fayazi and Hasani (17). Park et al. (21) similarly found that r-BAS Fun seeking could only predict internet addiction through the complete mediation of impulsivity. Other researchers have also found that individuals with internet addiction exhibit characteristics of high BAS and low self-control (9, 19). It may be attributable to individuals with high BAS, when faced with rewarding situations, are prone to actively pursuing short-term rewards, neglecting potential long-term negative consequences, leading to a decrease in self-control (34, 35). The decrease in self-control further results in a loss of control over behavior, immersing individuals in the online world and making it difficult to break free from internet addiction (9–11, 36).

r-BIS and FFFS could both directly predict internet addiction and also predict it through the mediation of self-control, confirming that r-BIS and FFFS could predict internet addiction through a partial mediation of self-control. Similar to this study, Fayazi and Hasani (17) found that r-BIS and FFFS could predict internet addiction through the partial mediation of anxiety and depression. Research has also shown that whether based on o-RST or r-RST, both subsystems can predict self-control (25). Moreover, self-control was a predictive factor of internet addiction (29).

Taken together, self-control played a mediating role in the relationship between r-BIS or FFFS and internet addiction among college students. Some scholars argue that individuals with high FFFS sensitivity are more responsive to aversive stimuli, making them more sensitive to stress in real life and more prone to experiencing anxiety and depression. The internet provides them a space to escape these negative emotions (19, 37). Similarly, individuals with high r-BIS are more likely to focus on the negative aspects and have more pessimistic expectations about outcomes (38). This conflicting tension between pessimistic expectations and future demands acts as a potential threat, quickly activating r-BIS (16, 28). Faced with threats, individuals may adopt compensatory measures, such as seeking pleasurable experiences through internet use for individuals with internet addiction. Over time, this weakens their ability to control internet use, making it more challenging to sustain goal-oriented healthy behavior (38, 39).

This study confirmed the immediate and long-term effects of reinforcement sensitivity on internet addiction and the mediating role of self-control in their causal relationship. Additionally, the results indicated differences in how each subsystem of reinforcement sensitivity predicted internet addiction. Specifically, r-BAS could only impact internet addiction through self-control, both immediately and in the long term. At the same time, r-BIS and FFFS could also have a direct impact through separate pathways. These results suggested that the predictive effect of r-BAS on internet addiction is weaker compared to r-BIS and FFFS. It provided evidence that, to some extent, relative to the rewarding drive for online behavior, internet addiction is more likely to be an avoidant choice in response to real-life anxiety and adverse events.

In conclusion, this study offers longitudinal empirical evidence for r-RST, further revealing the underlying mechanisms through which reinforcement sensitivity influences internet addiction. It also has implications for clinical interventions. In addition to directly intervening in reinforcement sensitivity, enhancing self-control in individuals with internet addiction might reduce the impact of reinforcement sensitivity, especially r-BAS, on internet addiction.

The study has some limitations. Firstly, it only examined the impact of reinforcement sensitivity on internet addiction. There might be bidirectional influences between personality and psychological abnormalities (17, 40). Research indicated that internet use can influence an individual’s sensitivity to gains and losses (19). Future research should focus on the bidirectional interaction between reinforcement sensitivity and internet addiction to gain a deeper understanding of the underlying mechanisms of internet addiction. Secondly, the study only focused on college students, and other groups, such as adolescents, are also worth investigating. Additionally, despite the sample size being similar to previous studies (21, 23, 25), it does not strictly qualify as a large-sample study, limiting the generalizability and interpretative power of the results. The small sample might also be a reason for the observed weak mediation effects. Therefore, the results need further validation in larger samples.

## Data availability statement

The raw data supporting the conclusions of this article will be made available by the authors, without undue reservation.

## Ethics statement

The studies involving humans were approved by Central China Normal University, Ethic Committe, EC, Institution Review Board. The studies were conducted in accordance with the local legislation and institutional requirements. The participants provided their written informed consent to participate in this study.

## Author contributions

JX: Writing – original draft. ZL: Data curation, Writing – original draft. WZ: Conceptualization, Writing – review & editing. WL: Investigation, Writing – original draft. LL: Writing – original draft. ZZ: Writing – original draft.
